# Enhancing Physician Satisfaction and Patient Safety Through an Artificial Intelligence–Driven Scheduling System in Anesthesiology

**DOI:** 10.31486/toj.24.0104

**Published:** 2025

**Authors:** William D. Sumrall, Jakob V. Oury, George M. Gilly

**Affiliations:** ^1^ Department of Anesthesiology, Ochsner Clinic Foundation, New Orleans, LA; ^2^ The University of Queensland Medical School, Ochsner Clinical School, New Orleans, LA

**Keywords:** *Anesthesia*, *artificial intelligence*, *burnout–professional*, *burnout–psychological*, *organizational innovation*, *personnel staffing and scheduling*, *safety*

## Abstract

**Background:**

Overcoming challenges to effective clinical practice depends on finding dynamic solutions to issues such as physician burnout and patient safety. This study evaluated the impact of an artificial intelligence (AI)–driven scheduling system on physician burnout and patient safety, using intraoperative transitions of care as an operative metric for patient safety.

**Methods:**

In May 2019, the Department of Anesthesiology at Ochsner Health in New Orleans, Louisiana, implemented an AI-driven scheduling system called Lightning Bolt Scheduling (PerfectServe, Inc). Utilizing an idealized design framework, the department steering committee analyzed 12 months of historic operating room data and developed more than 400 scheduling rules to optimize staffing. The scheduling rules, representing the steering committee's new work model, were provided as inputs into Lightning Bolt Scheduling, which then used combinatorial optimization to recommend an ideal staff schedule. Preimplementation and postimplementation data were collected on physician satisfaction, vacation approval rates, and intraoperative transitions of care.

**Results:**

Six months postimplementation, physician satisfaction scores and vacation approvals increased, reflecting improved work-life balance, schedule flexibility, and decreased symptoms of burnout. Additionally, 1,072 fewer handoffs occurred, equating to 71.5 fewer adverse events and a savings of approximately $335,550 in health care costs during the 21 months after implementation.

**Conclusion:**

Our study findings underscore the potential of data-driven scheduling systems to enhance physician well-being and patient safety, thereby promoting continuous improvement and adaptability in health care operations.

## INTRODUCTION

The modern health care landscape presents challenges to physicians, including burnout, work-life imbalance, job dissatisfaction, and risks to patient safety.^1,2^ To address these issues, innovative technologies such as artificial intelligence (AI) offer promising solutions.^3^

### Relationship of Physician Burnout to Scheduling

Physician burnout is often exacerbated by stressors related to suboptimal scheduling of health care operations, resulting in long hours, lack of flexibility, and the inability to balance work and free time.^4^ Such factors can induce chronic fatigue and negatively impact both work and family life.^4^ The review of physician burnout by Patel et al suggests that burnout can result in depression, substance use, and suicidal ideation in physicians and residents.^5^ Patel et al argue that spending time away from work is a potential solution, highlighting the importance of vacation time.^5^ Other research indicates a negative association between physician burnout and quality of care, with burnout associated with an increased risk of medical errors and adverse patient outcomes.^2^

### Relationship of Intraoperative Transitions of Care to Patient Safety

Intraoperative transitions of care—handoffs from one anesthesiologist to another during a surgical procedure—are common occurrences, with reported rates ranging from 2% to 40%.^6^ A 2024 study at a large academic hospital reported a transition of care rate of 40.4%.^6^ Until 2014, evidence that intraoperative transitions of care put patients at risk was limited; however, a negative association between surgical outcomes and the number of anesthesia providers per case has since been established.^6,7^ In 2014, Saager et al reported the results of a retrospective medical records review of 138,932 adult surgical patients at Cleveland Clinic that showed intraoperative transitions of anesthesia care led to increased hospital mortality and morbidity.^7^ In 2016, Hyder et al reviewed a cohort of 927 patients undergoing elective colorectal surgery and concluded that “care by additional attending anesthesiologists and in-room providers was independently associated with increased odds of postoperative complications.”^8^ In a retrospective cohort study of 313,066 adults undergoing major surgery that was published in 2018, Jones et al showed that for every 15 patients exposed to a transition of care event, 1 additional patient would be expected to experience a harm event (all-cause mortality, readmission, or a major postoperative complication within 30 days of surgery).^9^ Saha and Segal reviewed 121,077 cases of noncardiac surgery and reported in 2024 that intraoperative transitions of care were significantly associated with adverse outcomes.^6^

Transitions of care during surgery increase the likelihood of delays, miscommunication between providers, and patient harm.^7^ Intraoperative transitions of care can therefore serve as a quantitative, schedule-related factor that represents patient safety.

### Department of Anesthesiology Scheduling—Manual vs an Artificial Intelligence-Driven System

#### Manual Schedule Development.

Staff schedules for the Department of Anesthesiology at Ochsner Health in New Orleans, Louisiana, were developed using hand-typed Excel (Microsoft Corporation) documents. A team of 3 schedulers required an entire month to develop the next month's schedule, and no procedure was in place to effectively process an individual's schedule preferences. The process of developing a staff schedule yielded a suboptimal outcome that required a great deal of work on the part of the anesthesiology staff. Another issue with the manual system was the lack of capacity for optimizing intraoperative transitions of care.^3^

#### Lightning Bolt Scheduling System.

To address these scheduling challenges, anesthesiologists at Ochsner Health developed an organizational framework to optimize employee satisfaction and patient safety with the assistance of an AI-driven scheduling system called Lightning Bolt Scheduling (PerfectServe, Inc). Lightning Bolt Scheduling uses combinatorial optimization to develop shift schedules based on user-defined constraints and preferences. Combinatorial optimization is a mathematical process that evaluates a vast number of possible variable combinations to find the optimal solution, while balancing multiple constraints and objectives. The user defines scheduling rules that act as hard or soft constraints and incur penalties when the rules are broken. The severity of the penalty depends on the priority level assigned to each rule. The software then evaluates different scheduling configurations, seeking to minimize the total penalties by adhering to the highest priority rules while balancing other, less critical constraints. Lightning Bolt Scheduling explores more variations than can be manually assessed, quickly identifying the schedule with the lowest total penalty score.

For example, a high-priority rule may be the equal distribution of work hours averaged across a month. A higher penalty would be incurred if the work hour distribution rule were violated compared to a lower-priority rule, such as a staff member's preference for a certain assignment on Tuesdays. In practice, Lightning Bolt Scheduling allows lower-priority rules such as staff preferences to be broken more frequently than high priority rules such as work hour equalization to optimize the overall schedule. The result is a schedule that minimizes total penalties, balancing the adherence to user-defined rules with the need for efficiency. The Lightning Bolt Scheduling process is faster and more efficient than manual methods and creates a fair and flexible schedule that can meet the needs of staff members and potentially minimize the risk to patients.

#### Potential of Artificial Intelligence to Address Schedule-Related Challenges.

While AI applications are still in the early stages, AI has been used to process patient medical records, analyze quantitative data, and develop diagnostic prediction algorithms.^10^ Studies of AI-driven patient scheduling systems demonstrate the crucial ability to adapt to real-time changes such as emergency case additions.^3^ Maleki Varnosfaderani et al recommend further research into the use of AI in enhancing patient outcomes and system efficiency through application to administrative tasks.^3^ While the effect of AI-driven patient scheduling systems on patient satisfaction has been studied, to our knowledge, the effect of AI-driven physician shift scheduling on physician satisfaction or patient safety has not been studied, highlighting a gap in research.^3^

### Hypothesis

We hypothesized that the adoption of Lightning Bolt Scheduling would lead to improvements in metrics representing physician satisfaction and patient safety. By exploring the effectiveness of Lightning Bolt Scheduling, we aim to contribute insights to the ongoing dialogue on health care innovation and quality improvement through AI.

## METHODS

### Applying an Idealized Design Framework

To optimize physician satisfaction and patient safety, we applied an idealized design framework to envision optimal scheduling conditions.^11^ A deliberation process began by forming a steering committee composed of anesthesiologists from the 7 Ochsner Health anesthetic groups: pediatrics, pediatric cardiovascular, adult cardiovascular, regional, liver, core, and obstetrics. The steering committee held weekly meetings to outline preferences for working conditions and define a new department work model that would provide the inputs for Lightning Bolt Scheduling to optimize. The committee analyzed 2018 operating room volume data to (a) determine the desired length of an average work week, (b) assess staffing needs, and (c) develop daily staffing blocks to ensure adequate coverage with schedule predictability. This approach provided structure for Lightning Bolt Scheduling to use for schedule generation.

### Determining Staffing Needs for Lightning Bolt Scheduling Inputs

The steering committee determined that optimal scheduling would result in a 42.5-hour work week averaged over 4 weeks. Next, the steering committee used the 2018 operating room usage data to confirm that an average of 24 anesthesiology department staff members are needed to start each day. The next step was to ascertain how to allocate staff to staffing blocks. Table 1 shows the staffing blocks with the number of assigned staff members per time block. Work hours, staff numbers, and staffing blocks constituted the first data inputs needed for Lightning Bolt Scheduling to develop an optimized schedule.

**Table 1.** Department of Anesthesiology Staffing Blocks

Time Block	Number of Staff Assigned
6:30am-12:30pm	4
6:30am-2:30pm	5
6:30am-4:00pm	11
6:30am-6:30pm	4
12:00pm-6:30pm	3
12:00pm-8:00pm	1
6:30pm night float	2
8:00pm night float	2

Note: The table shows the number of staff members assigned to each time block. These staffing blocks were inputs to the Lightning Bolt Scheduling system and were used for schedule development.

### Programming Lightning Bolt Scheduling

Lightning Bolt Scheduling was programmed with more than 400 scheduling rules that emphasized appropriate workload distribution, schedule predictability, and schedule flexibility and minimized intraoperative care transitions. Specifically, the rules included a wide array of constraints: hundreds of customized schedule preferences from individual physicians (eg, “Dr A prefers every other Thursday off, if not a holiday week.”), varied call schedules among multiple subspecialities, regional site staffing needs, variations in seasonal staff availability, and late-stay requirements. The steering committee and department members collaboratively determined the priority of these rules through discussion, presentations, and refinement. All scheduling rules were presented at departmental meetings and are downloadable in spreadsheet format for distribution and transparency. Scheduling rules apply to attending anesthesiologists overseeing operations at the Ochsner Health main campus and at regional campus sites such as the Elmwood location and Ochsner Baptist. Residents and certified registered nurse anesthetists have separate scheduling models that were not evaluated for this study.

Each scheduling rule was programmed into the Lightning Bolt Scheduling system and assigned a penalty for rule violation. If a staff member joins or leaves the department, the system is unaffected because of the comprehensive rules and large staff size. However, the 3-person scheduling team can change or deactivate individual preference rules by submitting a request. If a new rule is needed, the scheduling team writes it into Lightning Bolt Scheduling. The scheduling team is responsible for maintaining proper scheduling and regularly reporting any major changes. Major changes are reviewed and vetted by the department's anesthesiologists.

The Ochsner Health Department of Anesthesiology transitioned to the Lightning Bolt Scheduling AI-driven scheduling system in May 2019. The entire process took 6 months to complete because the transition was used as a catalyst for a complete department overhaul. The schedule development process is now semiautomated to the degree that the task of schedule building can be completed with greater expediency compared to the previous manual system.

### Defining Performance Measures for Assessing Impact

To assess the impact of the Lightning Bolt Scheduling system on improving physician satisfaction, we used 2 performance measures. Physician satisfaction ratings were collected through unblinded satisfaction surveys conducted before and 6 months after implementation of Lightning Bolt Scheduling. The exact survey methodology cannot be reported because of copyright protections, but survey topics included perception of schedule flexibility and predictability, social support availability, work-life balance, and symptoms of burnout. A composite score of 1 to 5 was aggregated based on individual responses, with 5 representing the highest level of satisfaction. Scores were averaged across participants to compare preimplementation and postimplementation survey scores. The second performance measure was vacation approval and denial statistics compiled by Lightning Bolt Scheduling for the years 2018, 2019, and 2020.

Intraoperative transitions of care were identified as the schedule-related metric for patient safety. Transitions of care were defined as an instance of 2 anesthesiologists signed in during a case, and the transition of care rate was calculated as the total number of transitions divided by the total number of cases. Data collection spanned from 6 months preimplementation (November 2018 through April 2019) to monthly postimplementation pre-COVID and post-COVID (May 2019 through February 2020, and May 2020 through March 2021, respectively). A 2-sample z-test was used to compare the proportions of intraoperative transitions of care before and after Lightning Bolt Scheduling implementation because of the large sample size and independence between samples.^12^ The null hypothesis posited no difference between the previous manual scheduling system and the new AI-driven system. Intraoperative transitions of care were analyzed to quantify the impact on patient safety by using the finding in Jones et al that every 15 transitions of care equals 1 harm event.^9^ The Haidar et al finding that 1 harm event equates to a hospital-incurred cost of $4,693 was used to provide further context to the potential savings provided by the Lightning Bolt Scheduling system.^13^

## RESULTS

The findings show improvements in physician satisfaction, increased vacation day approvals, decreased transitions of care, and cost savings.

### Physician Satisfaction Scores

As shown in Table 2, physician satisfaction survey scores increased from 3.3 before Lightning Bolt Scheduling implementation to 4.2 postimplementation. The increase in satisfaction scores demonstrated quantitative improvements over the previous scheduling system, particularly in the areas of physician control and flexibility of scheduling, social support in the department, work-life balance, and symptoms of burnout.

**Table 2.** Physician Satisfaction Scores Before and After Implementation of the Lightning Bolt Scheduling System

Survey Time Point	Mean Score
Preimplementation	3.3
Postimplementation	4.2

Note: Satisfaction with schedule flexibility and predictability, social support availability, work-life balance, and symptoms of burnout was ranked on a scale of 1 to 5, with 5 representing the highest level of satisfaction.

### Vacation Day Approvals and Denials

Granted vacation days increased every year, from 1,424 days in 2018 (preimplementation), to 1,517 days in 2019, and 1,935 days in 2020 (Table 3). The vacation day denial rate decreased by 82% from 2018 to 2020, without requiring added staff to cover denials.

**Table 3.** Vacation Day Approvals and Denials by Year

Year	Vacation Days Granted	Vacation Days Denied
2018	1,424	39
2019	1,517	16
2020	1,935	7

Note: The Lightning Bolt Scheduling system was implemented in May 2019.

### Intraoperative Transitions of Care

During the 6 months before implementation of Lightning Bolt Scheduling, 2,628 intraoperative transitions of care were recorded for 25,614 cases, yielding a transition of care rate of 10.3% (Table 4). Overall, after implementation of Lightning Bolt Scheduling, transitions of care significantly decreased to 7,776 transitions in 85,905 cases, providing a postimplementation transition of care rate of 9.1% (z=5.82; *P*<0.001).

**Table 4.** Transitions of Care Analysis Before and After Implementation of the Lightning Bolt Scheduling System

Variable	Preimplementation^a^	Postimplementation^b^
Total cases, n	25,614	85,905
Transitions of care, n (transition of care rate, %)	2,628 (10.3)	7,776 (9.1)^c^
Fewer transitions of care, n^d^		1,072
Fewer harm events, n^e^		71.5
Savings, US dollars^f^		335,550

^a^Preimplementation is the 6-month period (November 2018 through April 2019) prior to implementation of the Lightning Bolt Scheduling system.

^b^Postimplementation is the pre-COVID period of May 2019 through February 2020 and the post-COVID period of May 2020 through March 2021.

^c^*P*<0.001.

^d^Fewer transitions of care was calculated by multiplying the total number of cases postimplementation (85,905) by the preimplementation transition of care rate (10.3%) to estimate handoffs without the Lightning Bolt Scheduling system in an equated case load (8,848) and then subtracting the number of postimplementation transitions of care (7,776): 85,905 × 0.103 = 8,848 – 7,776 = 1,072.

^e^Fewer harm events was calculated by dividing the fewer transitions of care number (1,072) by 15 in accordance with the Jones et al finding that for every 15 patients exposed to a transition of care event, 1 additional patient would be expected to experience a harm event.^9^

^f^The hospital-incurred cost of $4,693 per harm event reported in a 2023 study by Haidar et al^13^ was used to calculate the estimated savings resulting from the reduction in harm events: 71.5 × $4,693 = $335,550.

Month-to-month changes in the transition of care rate are shown in the Figure and in Table 5. The transition of care rate decreased to an average monthly rate of 8.76% at 10 months postimplementation (Table 5). The average monthly transition of care rate for the period May 2020 through March 2021 was 9.34% (Table 5).

**Figure.**
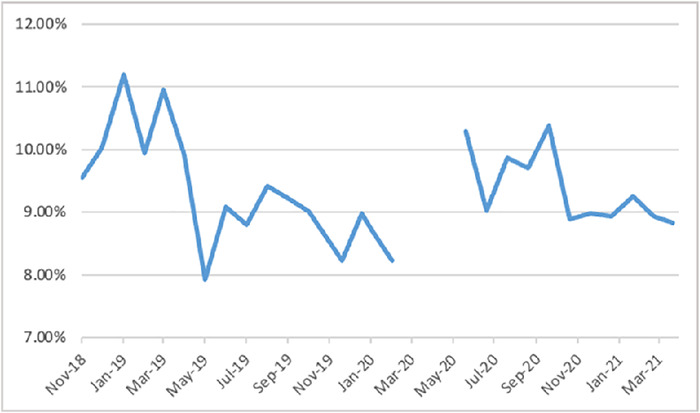
Intraoperative transition of care rate by month, November 2018 to February 2020 and May 2020 to March 2021.

**Table 5.** Monthly Cases, Transitions of Care, and Transition of Care Rate Before and After Implementation of the Lightning Bolt Scheduling System

Year/Month	Total Cases, n	Transitions of Care, n	Transition of Care Rate, %
2018			
November	4,636	443	9.56
December	4,024	404	10.04
2019			
January	4,488	502	11.19
February	4,192	417	9.95
March	4,009	439	10.95
April	4,265	423	9.92^a^
May	4,567	362	7.93
June	4,097	372	9.08
July	4,386	386	8.80
August	4,482	422	9.42
September	4,134	381	9.22
October	4,505	406	9.01
November	4,097	337	8.23
December	4,109	369	8.98
2020			
January	4,405	384	8.72
February	3,892	320	8.22^b^
May	2,517	259	10.29
June	3,559	321	9.02
July	4,275	422	9.87
August	3,998	388	9.70
September	4,150	431	10.39
October	4,137	368	8.90
November	3,917	352	8.99
December	4,223	377	8.93
2021			
January	3,720	344	9.25
February	3,764	336	8.93
March	4,971	439	8.83^c^

^a^The 6-month (November 2018 through April 2019) average transition of care rate before implementation of the Lightning Bolt Scheduling system = 10.3%.

^b^The rolling average transition of care rate for the pre-COVID period after implementation of the Lightning Bolt Scheduling system (May 2019 through February 2020) = 8.76%.

^c^The rolling average transition of care rate for the post-COVID period after implementation of the Lightning Bolt Scheduling system (May 2020 through March 2021) = 9.34%.

### Changes in Number of Transitions of Care, Harm Events, and Costs

Based on the data in Table 4, we estimated 1,072 fewer intraoperative transitions of care. To calculate the number, we multiplied the total number of cases postimplementation (85,905) by 10.3% (the preimplementation transition of care rate) to estimate the number of transitions of care (8,848) before the Lightning Bolt Scheduling system was implemented in an equated case load. From that number, we subtracted the postimplementation transitions of care (7,776): 85,905 × 0.103 = 8,848 – 7,776 = 1,072 fewer transitions of care. We divided 1,072 by 15 to determine the number of harm events.^9^ The result was 71.5 fewer harm events.^9^

Based on a harm event average cost of $4,693,^13^ Lightning Bolt Scheduling implementation saved an estimated $335,550 (Table 4).

## DISCUSSION

The implementation of an AI-driven scheduling system in the Department of Anesthesiology at Ochsner Health enhanced both physician satisfaction and patient safety.

### Addressing Physician Satisfaction

Central to our investigation was the objective of improving physician satisfaction. The adoption of Lightning Bolt Scheduling resulted in an increase in physician engagement scores and a consistent increase in vacation request approvals. The increase in physician satisfaction scores reflects the optimized scheduling efficiency and flexibility that contributed to decreasing symptoms of burnout. While our inability to provide visibility into the survey methodology and response quantity because of copyright restrictions prevents us from objectively demonstrating statistical significance, subjective and anecdotal observations suggest enhanced engagement and job satisfaction among physicians following the implementation of AI-driven scheduling, highlighting the potential value of our approach in fostering a positive work environment. Our reporting of improved physician satisfaction is intended to encourage other departments to replicate this study with more robust methodologies and improved data collection, as noted in the limitations section. By facilitating increased vacation acceptance rates, Lightning Bolt Scheduling provided the physical and mental benefit of spending time away from work.^5^

### Improving Patient Safety and Reducing Costs

Prioritizing patient safety, particularly in the context of continuity of care, was a key focus of schedule optimization. The 2-sample z-test showed that the difference in the intraoperative transitions of care rate between the preimplementation and postimplementation periods (10.3% vs 9.1%, respectively) was statistically significant (*P*<0.001). The AI-driven scheduling system likely contributed to this meaningful reduction in transitions of care, which could indicate improved continuity of care in the operating room. The reduction in intraoperative transfers of care postimplementation reflects a quantifiable improvement in patient safety, possibly decreasing the risk of miscommunication, delays in care, and patient harm that can accompany intraoperative handoffs.^7^ The decrease in harm events reduces costs.

### Study Limitations

The greatest limitation of the study is our inability to provide physician satisfaction survey methodology and results details beyond aggregate scores, affecting the reproducibility and significance of the study. In addition, the anesthesiology staff was aware of the transition to the AI-driven scheduling system, so their responses may have been positively influenced by the lack of blinding. Lack of blinding can introduce response bias, as participants may adjust their feedback based on their expectations or perceptions of the intervention, potentially skewing the results.^14^ The generalizability of findings is also limited as this study involved only the Department of Anesthesiology at Ochsner Health; the sample sizes and diversity may not capture variations in practices or patient populations in other health care settings. The time frame of data collection is potentially susceptible to confounding factors such as fluctuations and seasonal variations in workload and vacation trends.

### Recommendations for Future Research

Future research is needed to validate study findings across multiple institutions to assess the robustness and generalizability of these outcomes. Internal validity and generalizability can be enhanced by providing survey question details, mean or median scores for each survey question, the response rate, and the number of total completed surveys to allow for significance testing. Implementing blinded comparisons, adding multiple survey opportunities postimplementation, and expanding research to encompass a broader spectrum of medical departments would also improve the strength of findings. Furthermore, considerations of seasonal workload variations and efforts to mitigate potential biases are necessary to enhance outcome applicability. However, before investigating the use of AI in health care, researchers should consider the ethical, technological, regulatory, and social barriers to AI implementation—such as conflicts of interest, program usability and integration, patient data safety, and health care bias—that may apply to their institution.^15^

## CONCLUSION

Our adoption of an AI-driven scheduling system resulted in a quantifiable improvement in physician satisfaction and patient safety in our health care setting. By addressing key contributors to physician burnout such as schedule inflexibility and work-life imbalance and by improving continuity of care, we demonstrated that an AI-driven scheduling system improved metrics that indicated enhanced physician satisfaction and patient safety. Continued research and development efforts are vital for maximizing the viability of AI technology for the evolving needs of health care delivery.

## Notes

*This article meets the Accreditation Council for Graduate Medical Education and the American Board of Medical Specialties Maintenance of Certification competencies for Patient Care, Interpersonal and Communication Skills, Systems-Based Practice, and Practice-Based Learning and Improvement.*
